# Testing the fecundity advantage hypothesis with *Sitobion avenae*, *Rhopalosiphum padi*, and *Schizaphis graminum* (Hemiptera: Aphididae) feeding on ten wheat accessions

**DOI:** 10.1038/srep18549

**Published:** 2015-12-18

**Authors:** Xiang-Shun Hu, Xiao-Feng Liu, Thomas Thieme, Gai-Sheng Zhang, Tong-Xian Liu, Hui-Yan Zhao

**Affiliations:** 1 State Key Laboratory for Crop Stress Biology in Arid Areas, Key Laboratory of Crop Pest Management on the Northwest Loess Plateau of Ministry of Agriculture, College of Plant Protection, Northwest A&F University, No. 3, Weihui Road, Yangling, Shaanxi 712100, China; 2 BTL Bio–Test Labor GmbH Sagerheide, Birkenallee 19 D–18184, Sagerheide, Germany; 3 College of Agronomy, Northwest A&F University, No. 3, Weihui Road, Yangling, Shaanxi 712100, China

## Abstract

The fecundity advantage hypothesis suggests that females with a large body size produce more offspring than smaller females. We tested this hypothesis by exploring the correlations between life-history traits of three aphid species feeding on ten wheat accessions at three levels of analysis with respect to the host plant: overall, inter-accession, and intra-accession. We found that fecundity was significantly correlated with mean relative growth rate (MRGR), weight gain, and development time, and that the faster aphid develops the greater body and fecundity, depending on aphid species, wheat accession, and analyses level. Larger aphids of all three species produced more offspring overall; this held true for *Sitobion avenae* and *Schizaphis graminum* at the inter-accession level, and for *S. avenae*, *Rhopalosiphum padi,* and *S. graminum* for three, five, and eight accessions respectively at the intra-accession level. Only one correlation, between intrinsic rates of natural increase (r_m_) and MRGR, was significant for all aphid species at all three analysis levels. A more accurate statement of the fecundity advantage hypothesis is that cereal aphids with greater MRGR generally maintain higher r_m_ on wheat. Our results also provide a method for exploring relationships between individual life-history traits and population dynamics for insects on host plants.

Essential biological parameters for evaluating and understanding insect population dynamics include weight gain (WG), development time (DT), mean relative growth rate (MRGR), nymph survival rate, fecundity (F), and intrinsic rate of natural increase (r_m_)^1^,^2^,^3^,^4^,^5^,^6^,^7^,^8^,^9^,^10^,^11^. These life-history traits are generally used to evaluate the adaptability, phenotypic plasticity, and population dynamics of insect response to changes in environmental conditions and the resistance of host crop accessions to insects^12^,^13^,^14^,^15^,^16^,^17^,^18^,^19^,^20^,^21^,^22^. The fecundity advantage hypothesis, proposed by Darwin in 1874, suggests that large females have an evolutionary advantage over their smaller counterparts because they produce more offspring^23^,^24^. Ecologists use the correlations between various biological parameters to establish and interpret the relationships between individual life-history traits and population dynamics; correlations between F and other biological parameters have been extensively and exhaustively studied^1^,^2^,^3^,^4^,^5^,^6^,^7^,^8^,^9^,^10^,^11^,^24^,^25^,^26^,^27^,^28^,^29^,^30^,^31^.

Positive correlations between F and body weight or growth rate have been found in more than 60 insect species in eight orders—Coleoptera, Lepidoptera, Homoptera, Diptera, Ephemeroptera, Heteroptera, Hymenoptera, and Trichoptera^24^,^25^,^26^,^27^,^28^,^29^,^30^,^31^. Fenchel (1974) found a general correlation between r_m_ and average body weight in animals^31^. Insect adult body size has been used for predicting age at maturity^32^ and population stability in a seasonally variable environment^9^, and for building insect population models to address pest monitoring and control^16^,^33^. However, variations in environmental conditions including food quality, host resistance to insects, and the interaction between the insect and the host plant could influence the insect’s morphological, physiological, behavioral, and phenological traits^33^,^34^,^35^,^36^,^37^.

For aphids (Hemiptera: Aphididae), the correlations between reproductive potential (F) and body size or body weight may not be so straightforward^30^. Either F or r_m_ were significantly negatively correlated with DT in each of three clonal lineages of the cotton aphid, *Aphid gossypii* living on six commercial cotton cultivars^38^ and in the pea aphid *Acyrthosiphon pisum* living on 12 species of legumes^39^. A negative exponential relationship between the number of large embryos and adult weight was found for the green peach aphid *Myzus persicae* living on the sugar beet *Beta vulgaris* and potato *Solanum tuberosum*^40^, and, later, more than 90 aphid species living on 120 different host plant species^41^. However, the black bean aphid *A. fabae* did not exhibit significant linear correlations between growth rate or body size and reproductive output^42^.

The English grain aphid *Sitobion avenae* (Fab.), bird cherry-oat aphid *Rhopalosiphum padi* L., and greenbug aphid *Schizaphis graminum* (Rondani), are three important pests of wheat [*Triticum aestivum* (L.); Gramineae] and other cereals worldwide. *Rhopalosiphum padi* is a polyphagous insect that shows alternation of hosts; its winter hosts are Rosaceae, and its summer hosts are Gramineae^43^. *Sitobion avenae* and *Schizaphis graminum* are oligophagous insects and their hosts are mainly Gramineae^44^. All three aphid species have short life cycles and breed readily. Thus, the aphid–wheat system is an ideal biological model with which to study the influence of variations in host resistance to pests and the fecundity advantage hypothesis.

Our previous research estimated life history parameters for these three aphid species feeding on ten wheat accessions with different levels of resistance to aphids, and explored the correlations of five biological parameters among aphid species. We found that the wheat resistance to aphids has effects on the correlations between life-history traits of these three aphid species^17^. In this study, we used the same aphid species and wheat accessions to investigate the effects of wheat pest resistance on seven correlations: between F and DT, WG, and MRGR; between r_m_ and DT, WG, and MRGR; and between WG and DT, all within an aphid species. We analyzed these effects at three levels: overall (all wheat accessions pooled), inter-accession (across accessions), and intra-accession (within an accession). Our goals were to test the fecundity advantage hypothesis; to partition overall aphid–wheat effects into the effects of host plant accession and aphid species on development, size, and population growth of aphids under standard laboratory conditions; and to establish a linkage between individual life-history traits and population dynamics for these insect species.

## Materials and Methods

### Aphid species and wheat accessions

The three aphid species were *S. avenae*, *R. padi*, and *S. graminum*; the ten winter wheat accessions were ‘Batis’, ‘Astron’, ‘Xanthus’, ‘Ww2730’, ‘Xiaoyan22’, ‘98–10–30’, ‘98–10–32’, ‘98–10–35’, ‘186 Tm’, and ‘Amigo.’ We show the genetic relationship among the accessions and their relative resistances to aphid species in Table 1.

### Data Collection

Our methods of sampling, dissection, and data collection and storage were in accordance to those described by Hu *et al.* (2013)^17^, using laboratory conditions of 20 ± 0.5 °C (day) and 18 ± 0.5 °C (night), a photoperiod of L16: D8 h, and 70 ± 10% relative humidity. Each combination of aphid species and wheat accession was one set of experiments; there were 30 sets of experiments in all, each with 30–31 replicates. One replicate consisted of a single first instar nymph transferred to a single seedling within 24 hours of birth. Five life-history traits were measured for each aphid individual: development time (DT), measured from birth to adult emergence +0.5 d; weight gain (WG), where WG = Wa − Wn, and Wa is adult weight within 24 hours of emergence and Wn is the weight of the first instar nymph 24 hours after birth; fecundity (F), the number of offspring produced per female within a time period equal to development time; mean relative growth rate (MRGR), where MRGR = (ln Wa − ln Wn)/DT; intrinsic rate of natural increase (r_m_), r_m_ = 0.738 × ln (F)/DT^17^,^45^,^46^,^47^,^48^. If any of the five parameters for an individual aphid were missing from the data set, the replicate was excluded. Less than 1% of *S. avenae* and *S. graminum* and 16.43% of *R. padi* were alataes, and because the biological parameters are different between apterae and alatae, all alatae data were excluded as well.

### Data Analysis

We analyzed seven relationships between life-history parameters: between F and WG, MRGR, and DT; between r_m_ and WG, MRGR, and DT; and between WG and DT for each aphid species on three levels (overall, inter-accession, and intra-accession). At the overall level, analyses were performed with parameter values for individual replicates with no consideration of wheat accession. There were 287 replicates for *S. avenae*, 234 for *R. padi*, and 221 for *S. graminum*. At the inter-accession level, analysis was performed using the mean values for each parameter for each aphid species on each wheat accession; there were 10 samples per aphid species. At the intra-accession level, analysis used individual aphid data for each of the 30 unique combinations of aphid species and wheat accession. After excluding samples because of missing data or alatae status, there were 26–31 aphid replicates per accession for *S. avenae,* 17–31 replicates per accession for *R. padi,* and 15–25 replicates per accession for *S. graminum.*

### Analysis methods

We used SPSS version 17.0 to calculate Pearson’s correlation coefficients for the relationships between parameters. Because both r_m_ [=0.738 × ln (F)/DT] and MRGR [=(ln Wa − ln Wn)/DT] were calculated using DT, partial correlation coefficients between r_m_ and MRGR, controlled for DT, were also examined.

We used Sigmaplot 12.0 to draw scatterplots for pairs of parameters to compare the correlations among the three aphid species indirectly.

## Results

### Correlations between life-history parameters of aphid species

#### S. avenae

Table 2 presents the correlation coefficients between parameters of *S. avenae* at all three analysis levels.

At the overall level, F was significantly positively correlated with WG, MRGR, and DT; r_m_ was significantly positively correlated with WG and MRGR, but negatively correlated with DT; and WG was significantly negatively correlated with DT.

At the inter-accession level, F was significantly positively correlated with WG and MRGR; r_m_ was significantly positively correlated with WG and MRGR; and F, r_m_, and WG were not correlated with DT.

At the intra-accession level, there were significant correlations between F and DT, WG, and MRGR for five, three, and zero accessions respectively; there were significant correlations between r_m_ and DT, WG, and MRGR for five, ten, and ten accessions respectively; there were significant correlations between WG and DT for seven accessions.

#### R. padi

The correlation coefficients between parameters of *R. padi* are shown in Table 3.

At the overall level, F was significantly positively correlated with DT, WG, and MRGR; r_m_ was significantly positively correlated with WG and MRGR, and negatively correlated with DT; and WG was significantly negatively correlated with DT.

At the inter-accession level, F was not significantly correlated with DT, WG, or MRGR; r_m_ was positively correlated with WG and MRGR and significantly negatively correlated with DT; and WG was not significantly correlated with DT.

At the intra-accession level, there were significant correlations between F and DT, WG, and MRGR for one, five, and two accessions respectively; there were significant correlations between r_m_ and DT, WG, and MRGR for ten, six, and ten accessions respectively; there were significant correlations between WG and DT for only one accession.

#### S. graminum

Correlation coefficients between parameters of *S. graminum* are shown in Table 4.

At the overall level, F was positively correlated with WG and MRGR and significantly negatively correlated with DT; r_m_ was positively correlated with WG and MRGR and significantly negatively correlated with DT; and WG was significantly negatively correlated with DT.

At the inter-accession level, F and r_m_ were both significantly positively correlated with WG and MRGR; and F, r_m,_ and WG were all significantly negatively correlated with DT.

At the intra-accession level, there were significant correlations between F and DT, WG, and MRGR for four, seven, and eight accessions respectively; there were significant correlations between r_m_ and DT, WG, and MRGR for all ten accessions; there were significant correlations between WG and DT for all ten accessions.

### Comparison of aphid species based on their life-history correlations

#### Overall

**Figure 1:**
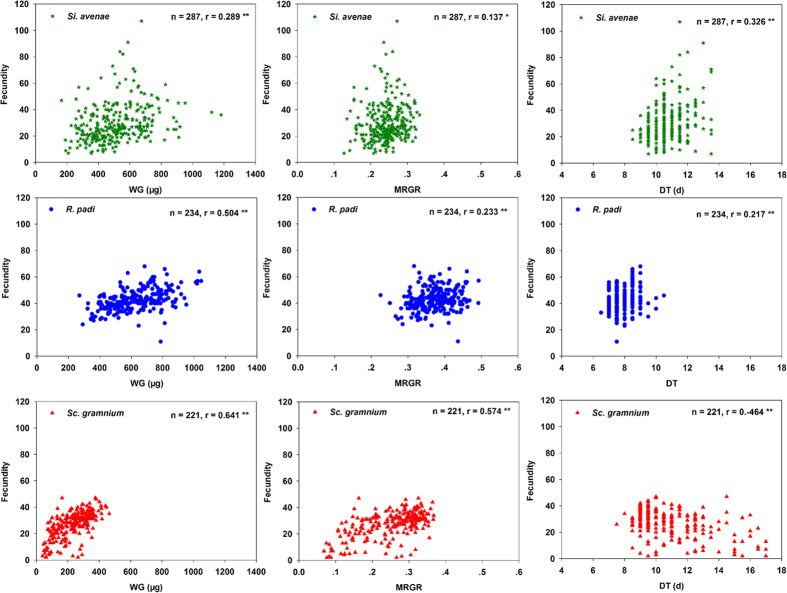
Fecundity (F) correlated with weight gain (WG), the mean relative growth rate (MRGR) and development time (DT) for three aphid species at the overall level.

**Figure 2:**
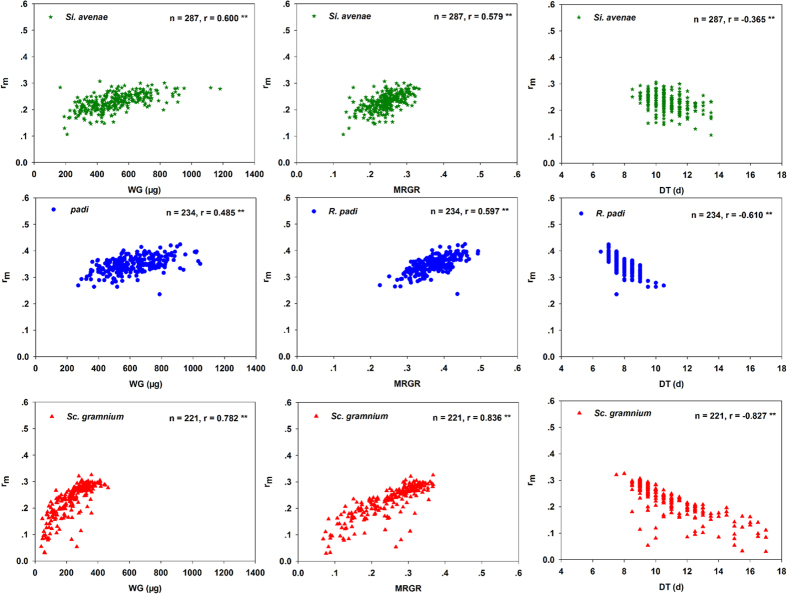
Intrinsic rates of natural increase (r_m_) correlated with weight gain (WG), mean relative growth rate (MRGR), and development time (DT) for three aphid species at the overall level.

**Figure 3:**
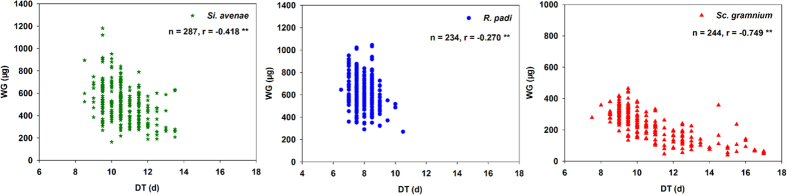
Weight gain (WG) correlated with development time (DT) for three aphid species at the overall level.

At the overall level, correlations for all seven life-history parameter pairs were significant for all three aphid species. Scatterplots of these data are shown in Figs 1, 2 and 3. Correlations between F and DT were strongly positive for *S. avenae* and *R. padi*, but strongly negative for *S. graminum*. F was significantly positively correlated with WG and MRGR for all three aphid species (Fig. 1). Correlations for r_m_ were significantly negative with DT, and significantly positive with WG and MRGR (Fig. 2) for all three aphid species. There were significant negative correlations between WG and DT for all three aphid species (Fig. 3).

#### Inter-accession

**Figure 4:**
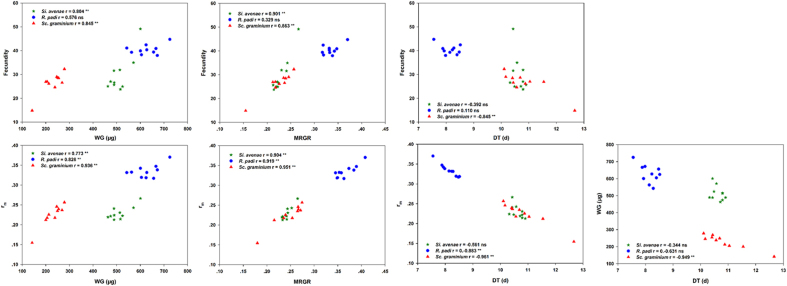
Intrinsic rates of natural increase (r_m_) correlated with weight gain (WG), mean relative growth rate (MRGR) and development time (DT) for three aphid species at the inter-accession level across ten wheat accessions.

Scatterplots for all seven life-history parameter correlations at the inter-accession level are shown in Fig. 4. F was significantly positively correlated with MRGR and WG for *S. avenae* and *S. graminum*, but not for *R. padi*. There was a positive correlation between r_m_ and both WG and MRGR for all three aphid species. The correlations between WG and DT were also significantly negative for all three species. The correlation between F and DT was a strongly negative correlation for *S. graminum*, not for *S. avenae* and for *R. padi*. The correlation between DT and r_m_ was significantly negative for *R. padi* and *S. graminum*, but not for *S. avenae*.

#### Intra-accession

**Figure 5:**
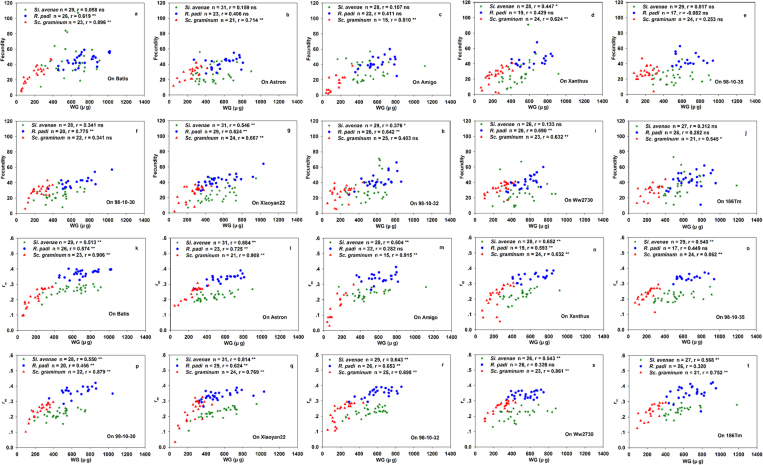
(**a**–**j**) Correlations between fecundity (**F**) and weight gain (WG) and (**k**–**t**) between intrinsic rates of natural increase (r_m)_ and weight gain (WG) at the intra-accession level within ten wheat accessions.

**Figure 6:**
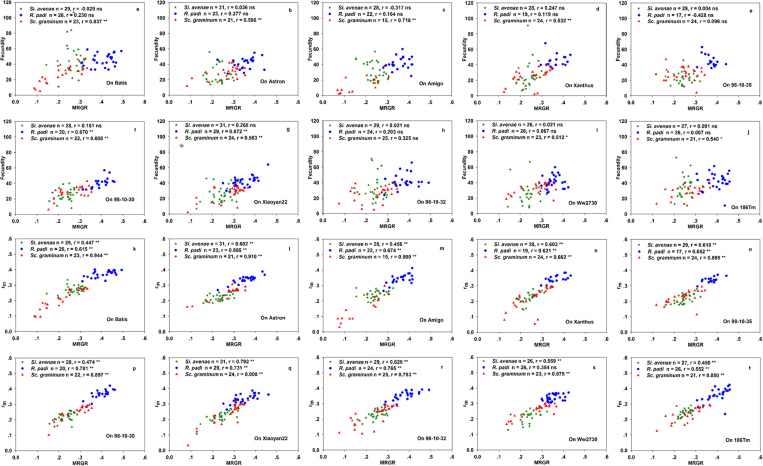
(**a**–**j**) Correlations between fecundity (**F**) and development time (DT), and (**k**–**t**) between intrinsic rates of natural increase (r_m_) and development time (DT) at the intra-accession level within ten wheat accessions.

**Figure 7:**
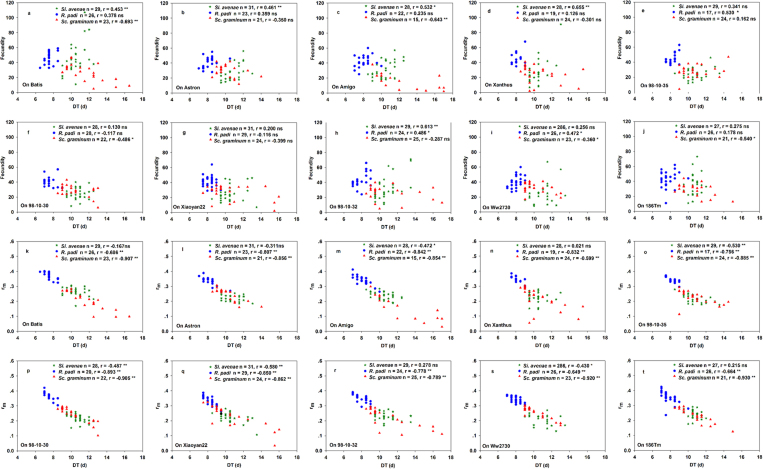
(**a**–**j**) Correlations between fecundity (**F**) and mean relative growth rate (MRGR), and (**k**–**t**) between intrinsic rates of natural increase (r_m_) and MRGR at the intra-accession level within ten wheat accessions.

Scatterplots of the correlations between aphid species life-history parameters for each of the ten wheat accessions are shown in Figs 5, 6 and 7, and the appendix table.

Correlations between life-history traits were different among the aphid species. There were significant correlations between F and WG for three accessions (‘Xanthus’, ‘Xiaoyan22’, and ‘98-10-30’) for *S. avenae*; for five accessions (‘Batis’, ‘98-10-30’, ‘Xiaoyan22’, ‘98-10-32’, and ‘Ww2730’) for *R. padi*; and for all accessions except ‘98-10-32’ and ‘98-10-35’ for *S. graminum* (Fig. 5a–j).

There were significant correlations between r_m_ and WG for all ten wheat accessions for *S. avenae* and *S. graminum*, and for the six accessions ‘Batis’, ‘Astron’, ‘Xanthus’, ‘98-10-30’, ‘Xiaoyan2’, and ‘98-10-32’ for *R. padi* (Fig. 5k–t).

F significantly correlated with MRGR for no accessions for *S. avenae*; for the two accessions ‘98-10-30’ and ‘Xiaoyan22’ for *R. padi*; and for all accessions except ‘98-10-32’ and ‘98-10-35’ for *S. graminum* (Fig. 6a–j). There were significant correlations between r_m_ and MRGR for *S. avenae* and *S. graminum* for all ten accessions, and in *R. padi* all accessions except ‘Ww2730’ (Fig. 6k–t).

There were significant correlations between F and DT for five accessions for *S. avenae* (‘Batis’, ‘Astron’, ‘Amigo’, ‘Xanthus’, and ‘98-10-32’), four accessions for *S. graminum* (‘Batis’, ‘Amigo’, ‘98-10-30’, and ‘186 Tm’), and three accessions for *R. padi* (‘98-10-35’, ‘98-10-32’, and ‘Ww2730’) (Fig. 7a–j). There were significant correlations between DT and r_m_ for all ten accessions for *R. padi* and *S. graminum*, and five accessions (‘Amigo’, ‘98-10-35’, ‘98-10-30’, ‘Xiaoyan22’, and ‘Ww2730’) for *S. avenae* (Fig. 7k–t).

## Discussion

### Correlations between fecundity and other biological parameters

Although F of most insect taxa increases with WG or body size^25^,^26^,^27^,^28^, we found that correlations between F and other biological parameters varied depending on aphid species, host wheat accession, the interaction between aphid species and host accession, and the level of the analyses (overall, inter-accession, or intra-accession). Previous work reported significant correlations between F and MRGR for *R. padi* at the overall level for five host species^49^; and between F and DT for three *A. gossypii* clonal lineages across six commercial cotton cultivars^38^; for *A. gossypii*, *Brevicoryne brassicae* (L.), and *R. padi* feeding on plants treated with sublethal doses of insecticides^50^,^51^,^52^; and for *S. avenae* feeding on wheat infected with barley yellow dwarf virus^53^. However, the significant correlation we found between F and DT for *S. avenae* did not agree with what Özder (2002)^54^ or Wojciechowicz–Zytko & van Emden (1995)^42^ reported.

These data indicate that larger aphids produced more offspring at the overall level for all three aphid species. At the inter-accession level, large *S. avenae* and *S. graminum* produced more offspring than small individuals did, but large *R. padi* did not produce more offspring than small *R. padi*. At the intra-accession level, whether larger aphids produced more offspring depended on the wheat accession on which they fed.

### Correlations between r_m_ and other biological parameters

That r_m_ significantly positively correlated with MRGR in nearly all cases in this study agrees with previous findings for *R. padi* at the overall level for five host plant species^49^ and for *A. fabae* with *V. faba* cultivars ‘Aquadulce’ and ‘Relon’ though not with seven other cultivars^42^. Our finding that r_m_ is significantly negatively correlated with DT agrees with previous reports for cotton aphid *A. gossypii* clonal lineages across six commercial cotton cultivars^38^ and for the pea aphid *A. pisum* on 12 species of legumes^39^.

The equations for MRGR and r_m_ both have a denominator of DT. To remove the effect of DT, we calculated the partial correlation coefficients that control for DT. We found that the partial correlation between r_m_ and MRGR was significant for *S. avenae*, *R. padi,* and *S. graminum* for five, five, and six wheat accessions respectively. This means the correlations between r_m_ and MRGR depended on both DT of aphids and wheat accession.

### The accessional resistance effect on the correlations

The host plant’s resistance to aphids can affect the aphid individual and population traits^10^,^14^,^15^,^16^,^17^,^18^,^19^,^20^,^34^,^38^,^46^,^47^. We found that accessional resistance has influence on the life-history traits of *S. graminum*. For example, of the ten wheat accessions, the ‘Amigo’ accession, which has a gene for resistance to *S. graminum* biotypes B and C^55^, had the lowest nymphal survival, WG, MRGR, F, and r_m_ and longest DT for this aphid^17^. These results are similar to those reported for hypersensitive apple trees that can rapidly necrose tissue at aphid feeding sites (a resistance reaction), which induced lower F and MRGR for the rosy apple aphid *Dysaphis plantaginea* compared to susceptible apple trees^56^. Accessional resistance did not have broad influence on the life-history traits of *S. avenae* and *R. padi*. For example, ‘98-10-30’, which is resistant to *S. avenae* due to a high level of hydroxamic acid^17^,^57^ had the lowest WG and MRGR for this aphid, but its F and r_m_ were not the lowest and DT not the highest. ‘Xiaoyan22’ has a gene for resistance to *R. padi*^17^; the WG was lowest but F was high. The correlations between aphid life-history traits could help define and differentiate the mechanisms of wheat accession resistance to different aphid species.

## Conclusion

In summary, the fecundity advantage hypothesis is not supported in the aphid–wheat systems studied. For these aphid species, larger aphids produce more offspring only at the overall level; for *S. avenae* and *S. graminum* this is also true at the inter-accession level, but not for *R. padi*. At the intra-accession level of analysis, we found that the resistance characteristics of wheat accessions significantly affect the correlations between aphid life-history traits that link the individual to the population. A more accurate statement is that aphids that are larger and develop more quickly generally maintain higher population growth rates.

The time period used to determine WG, MRGR, and DT was from nymphae birth to adult emergence, but that used for F and r_m_ was the entire lifespan. Host plants may become weak or die during the experiment in the laboratory, due to lack of fertilizer or constraints on root growth, leading to experimental failure. Based on our results, we conclude that one may use the parameters that can be determined in a short amount of time to calculate parameters that would need more time to be measured directly. For example, we can use WG to calculate r_m_ for *S. avenae*, use DT to calculate r_m_ for *R. padi*, and use DT or WG to calculate r_m_ for *S. graminum*. Our results also provide a method for exploring relationships between individual life-history traits and population dynamics for insects on host plants.

## Additional Information

**How to cite this article**: Hu, X.-S. *et al.* Testing the fecundity advantage hypothesis with *Sitobion avenae,* *Rhopalosiphum padi,* and *Schizaphis graminum* (Hemiptera: Aphididae) feeding on ten wheat accessions. *Sci. Rep.* **5**, 18549; doi: 10.1038/srep18549 (2015).

## Supplementary Material

Supplementary Information - Appendix table

## Figures and Tables

**Table 1:** Wheat accessions used^17^.

Wheat accession	Country of origin	Resistance to aphid	Genetic Relationship
Batis	Germany	*S. graminum* (+)	*T. aestivum*
Astron	Germany	*S. avenae* (++), *R. padi* (+)	*T. aestivum*
Xanthus	Germany	*R. padi*(+)	*T. aestivum*
Ww2730	Germany	*S. avenae* (++), *R. padi* (+)	*T. aestivum*
Amigo	USA	*S. graminum* (++), *R. padi* (+)	*T. aestivum* with an 1AL1RS wheat-rye (*Secale cereale*) chromosome translocation
98-10-30	China	*S. avenae* (++)	Hybrid of *T. aestivum* (Chirs) and *T. turgidum*
98-10-32	China	*S. avenae* (+)	Hybrid of *T. aestivum* (Chirs) and *T. turgidum*
98-10-35	China	*S. avenae* (+)	Hybrid of *T. aestivum* (Chirs) and *T. turgidum*
186 Tm	China	*S. avenae*, S. graminum*	Hybrid of *T. aestivum* and *T. monococcum*
Xiaoyan22	China	*R. padi* (+)	Hybrid of *T. aestivum* and *Agropyrum repens* Beauvois (*T. repens*)

Note: ‘++’ highly resistant, ‘+’ resistant.

*The survival of greenbug *S. graminum* and English gain aphid *S. avenae* were lowest on 186 Tm, which indicates that the segregation of resistance or susceptibility was not stably inherited in 186 Tm^17^.

**Table 2:** Correlation coefficients recorded for *S. avenae*.

Factors		Overall n = 287	Inter-accession n = 10	Intra-accession (number of accessions out of 10 with significant correlation)
F	DT	0.326**	−0.392	5
	WG	0.289**	0.804**	3
	MRGR	0.137*	0.901**	0
r_m_	DT	−0.365**	−0.561	5
	WG	0.600**	0.773**	10
	MRGR	0.579**(0.483**)	0.904**(0.871**)	10 (5)
WG	DT	−0.418**	−0.344	7

Note: ‘*’ indicates the correlation was significant at *p* < 0.05, ‘**’ indicates the correlation was significant at *p* < 0.01. ‘F’ is fecundity, ‘DT’ is development time, ‘WG’ is weight gain, ‘MRGR’ is the mean relative growth rate, ‘r_m_’ is the intrinsic rate of natural increase. The number in parentheses is the partial correlation coefficient controlling for DT. The notations in the following tables are the same.

**Table 3:** Correlation coefficients recorded for *R. padi*.

Factors		Overall n = 234	Inter-accession n = 10	Intra-accession (number of accessions out of 10 with significant correlation)
F	DT	0.217**	0.110	3
	WG	0.504**	0.576	5
	MRGR	0.233**	0.329	2
r_m_	DT	−0.610**	−0.883**	10
	WG	0.485**	0.826**	6
	MRGR	0.597**(0.348**)	0.919**(0.647)	9 (5)
WG	DT	−0.270**	−0.631	1

**Table 4:** Correlation coefficients recorded for *S. graminium*.

Factors		Overall n = 221	Inter-accession n = 10	Intra-accession (number of accessions out of 10 with significant correlation)
F	DT	−0.464**	−0.869**	5
	WG	0.641**	0.845**	8
	MRGR	0.574**	0.863**	8
r_m_	DT	−0.827**	−0.961**	10
	WG	0.782**	0.936**	10
	MRGR	0.836**(0.390**)	0.951**(0.269)	10 (6)
WG	DT	−0.749**	−0.949**	10
