# Supplementary material for: Testing the fecundity advantage hypothesis with Sitobion avenae, Rhopalosiphum padi, and Schizaphis graminum (Hemiptera: Aphididae) feeding on ten wheat accessions
Source: Sci Rep. 2015 Dec 18;5:18549. doi: 10.1038/srep18549 (PMC4683512; doi:10.1038/srep18549)
Supplement: Supplementary Information - Appendix table [file srep18549-s1.doc]

**Testing the fecundity advantage hypothesis with *Sitobion avenae*, *Rhopalosiphum padi*, and *Schizaphis graminum* (Hemiptera: Aphididae) feeding on ten wheat varieties**

Xiang-Shun Hu^1^, Xiao-Feng Liu^1^, Thomas Thieme^2^, Gai-Sheng Zhang^3^, Tong-Xian Liu^1, *^ & Hui-Yan Zhao^1, *^

1. State Key Laboratory for Crop Stress Biology in Arid Areas, Key Laboratory of Crop Pest Management on the Northwest Loess Plateau of Ministry of Agriculture, College of Plant Protection, Northwest A&F University, No. 22, Xinong Road, Yangling, Shaanxi 712100, China

2. BTL Bio–Test Labor GmbH Sagerheide, Birkenallee 19 D–18184, Sagerheide, Germany

3. College of Agronomy, Northwest A&F University, No. 22, Xinong Road, Yangling, Shaanxi 712100, China

Affiliations Contributions Competing financial interests Corresponding author

**Affiliations**

1. State Key Laboratory for Crop Stress Biology in Arid Areas, Key Laboratory of Crop Pest Management on the Northwest Loess Plateau of Ministry of Agriculture, College of Plant Protection, Northwest A&F University, No. 22, Xinong Road, Yangling, Shaanxi, 712100, China

Xiang–Shun Hu, Xiao–Feng Liu, Hui–Yan Zhao, Tong–Xian Liu

2. BTL Bio–Test Labor GmbH Sagerheide, Birkenallee, 19, D–18184, Sagerheide, Germany

Thomas Thieme

3. College of Agronomy, Northwest A&F University, No. 22, Xinong Road, Yangling, Shaanxi, 712100, China

Gai-Sheng Zhang

**Contributions**

X.-S. H., H.-Y. Z., T. T. designed research, X.-S. H. and H.-Y. Z., performed research, X.-S. H. and X.-F. L. analyzed data, prepared figure and wrote the paper; X.-S. H., X.-F. L. and T.-X. L. contributed to the interpretation of the results and reviewed the manuscript. G.-S. Z., T. T. and H.-Y. Z. provided wheat seeds. All authors reviewed the manuscript.

**Competing financial interests**

The authors declare no competing financial interests.

**Corresponding author**

* Tong-Xian Liu: [txliu@nwsuaf.edu.cn](mailto:txliu@nwsuaf.edu.cn); Hui-Yan Zhao: [zhaohy@nwsuaf.edu.cn](mailto:zhaohy@nwsuaf.edu.cn)

Appendix table: Correlation coefficients between parameters recorded for apterae of three aphid species reared on ten wheat varieties (Intra-variety)

| Aphid species | Wheat lines | Aphid No. | | F | | | | | | | |  | | | r_m_ | | | | | | | |  | | WG | |  | | Critical value | | |
| --- | --- | --- | --- | --- | --- | --- | --- | --- | --- | --- | --- | --- | --- | --- | --- | --- | --- | --- | --- | --- | --- | --- | --- | --- | --- | --- | --- | --- | --- | --- | --- |
|  |  |  |  | WG | | MRGR | | | DT | | |  |  |  | WG | | | | MRGR | | DT | |  |  | DT | |  |  | P = 0.05 | | P = 0.01 |
| *Si. avenae* | Batis | 29 | | 0.058 | | -0.029 | | | 0.453* | | |  | | | 0.513** | | | | 0.447**(0.275) | | -0.167 | |  | | -0.442* | |  | | 0.367(0.374) | | 0.471(0.479) |
|  | Astron | 31 | | 0.159 | | 0.036 | | | 0.461** | | |  | | | 0.684** | | | | 0.602**(0.505**) | | -0.311 | |  | | -0.553** | |  | | 0.355(0.361) | | 0.456(0.463) |
|  | Amigo | 28 | | 0.107 | | -0.317 | | | 0.532** | | |  | | | 0. 604** | | | | 0.456*(-0.100) | | -0.472* | |  | | -0.754** | |  | | 0.374(0.381) | | 0.479(0.487) |
|  | Xanthus | 28 | | 0.447* | | 0.247 | | | 0.655** | | |  | | | 0.652** | | | | 0.603**(0.605**) | | 0.021 | |  | | -0.049 | |  | | 0.374(0.381) | | 0.479(0.487) |
|  | 98-10-35 | 29 | | 0.017 | | 0.004 | | | 0.341 | | |  | | | 0.540** | | | | 0.610**(0.397*) | | -0.530** | |  | | -0.623** | |  | | 0.367(0.374) | | 0.471(0.479) |
|  | 98-10-30 | 28 | | 0.341 | | 0.181 | | | 0.130 | | |  | | | 0.550** | | | | 0.474*(0.323) | | -0.487** | |  | | -0.492** | |  | | 0.374(0.381) | | 0.479(0.487) |
|  | Xiaoyan22 | 31 | | 0.546** | | 0.268 | | | 0.200 | | |  | | | 0.814** | | | | 0.792**(0.705**) | | -0.580** | |  | | -0.480** | |  | | 0.355(0.361) | | 0.456(0.463) |
|  | 98-10-32 | 29 | | 0.376* | | 0.021 | | | 0.613** | | |  | | | 0.643** | | | | 0.625**(0.613**) | | -0.278 | |  | | -0.231 | |  | | 0.367(0.374) | | 0.471(0.479) |
|  | Ww2730 | 26 | | 0.133 | | 0.021 | | | 0.256 | | |  | | | 0.543** | | | | 0.559**(0.365) | | -0.430* | |  | | -0.489* | |  | | 0.388(0.396) | | 0.496(0.505) |
|  | 186tm | 27 | | 0.312 | | 0.091 | | | 0.275 | | |  | | | 0.568** | | | | 0.450*(0.305) | | -0.215 | |  | | -0.369 | |  | | 0.381(0.388) | | 0.487(0.497) |
| *R. padi* | Batis | | 26 | | 0.619** | | 0.230 | | | 0.378 |  | | | 0.574** | | | 0.615**(0.470*) | | | -0.686** | |  | | -0.095 | |  | | 0.388(0.396) | | 0.496(0.505) | |
|  | Astron | | 23 | | 0.406 | | 0.277 | | | 0.359 |  | | | 0.731** | | | 0.885**(0.768**) | | | -0.807** | |  | | -0.471** | |  | | 0.413(0.423) | | 0.526(0.537) | |
|  | Amigo | | 22 | | 0.411 | | 0.164 | | | 0.235 |  | | | 0.282 | | | 0.674**(0.351) | | | -0.842** | |  | | -0.086 | |  | | 0.423(0.433) | | 0.537(0.549) | |
|  | Xanthus | | 19 | | 0.429 | | 0.119 | | | 0.126 |  | | | 0.593** | | | 0.621**(0.275) | | | -0.832** | |  | | -0.388 | |  | | 0.456(0.468) | | 0.575(0.590) | |
|  | 98-10-35 | | 17 | | -0.082 | | -0.428 | | | 0.530* |  | | | 0.449 | | | 0.652**(0.042) | | | -0.756** | |  | | -0.417 | |  | | 0.482(0.497) | | 0.606(0.623) | |
|  | 98-10-30 | | 20 | | 0.775** | | 0.670** | | | -0.117 |  | | | 0.456* | | | 0.781**(0.768**) | | | -0.893** | |  | | -0.120 | |  | | 0.444(0.456) | | 0.623(0.575) | |
|  | Xiaoyan22 | | 29 | | 0.725** | | 0.672** | | | -0.116 |  | | | 0.624** | | | 0.731**(0.671**) | | | -0.850** | |  | | -0.325 | |  | | 0.367(0.374) | | 0.471(0.479) | |
|  | 98-10-32 | | 24 | | 0.642** | | 0.203 | | | 0.486* |  | | | 0.653** | | | 0.765**(0.655**) | | | -0.778** | |  | | -0.163 | |  | | 0.404(0.413) | | 0.515(0.526) | |
|  | Ww2730 | | 26 | | 0.690* | | 0.067 | | | 0.472* |  | | | 0.326 | | | 0.354(0.251) | | | -0.649** | |  | | 0.270 | |  | | 0.388(0.396) | | 0.496(0.505) | |
|  | 186tm | | 26 | | 0.282 | | 0.007 | | | 0.178 |  | | | 0.320 | | | 0.552**(0.102) | | | -0.664** | |  | | -0.273 | |  | | 0.388(0.396) | | 0.496(0.505) | |
| *S. gramnium* | Batis | 23 | | 0.896** | | 0.837** | | -0.693** | | | | |  | | | 0.906** | | 0.944**(0.740**) | | | -0.907** | |  | | -0.785** | |  | | 0.413(0.423) | | 0.526(0.537) |
|  | Astron | 21 | | 0.714** | | 0.586** | | -0.350 | | | | |  | | | 0.908** | | 0.910**(0.532*) | | | -0.856** | |  | | -0.764** | |  | | 0.433(0.444) | | 0.549(0.561) |
|  | Amigo | 15 | | 0.810** | | 0.718** | | -0.643** | | | | |  | | | 0.915** | | 0.900**(0.184) | | | -0.854** | |  | | -0.843** | |  | | 0.514(0.532) | | 0.641(0.661) |
|  | Xanthus | 24 | | 0.624** | | 0.532** | | -0.301 | | | | |  | | | 0.632** | | 0.662**(0.546**) | | | -0.599** | |  | | -0.682** | |  | | 0.404(0.413) | | 0.515(0.526) |
|  | 98-10-35 | 24 | | 0.253 | | 0.096 | | 0.162 | | | | |  | | | 0.862** | | 0.895**(0.544**) | | | -0.885** | |  | | -0.799** | |  | | 0.404(0.413) | | 0.515(0.526) |
|  | 98-10-30 | 22 | | 0.664** | | 0.608** | | -0.486* | | | | |  | | | 0.879** | | 0.897**(0.484*) | | | -0.905** | |  | | -0.834** | |  | | 0.423(0.433) | | 0.537(0.549) |
|  | Xiaoyan22 | 24 | | 0.667** | | 0.563** | | -0.399 | | | | |  | | | 0.769** | | 0.908**(0.657**) | | | -0.862** | |  | | -0.657** | |  | | 0.404(0.413) | | 0.515(0.526) |
|  | 98-10-32 | 25 | | 0.403 | | 0.325 | | -0.287 | | | | |  | | | 0.698** | | 0.783**(0.275) | | | -0.789** | |  | | -0.698** | |  | | 0.404(0.413) | | 0.515(0.526) |
|  | Ww2730 | 23 | | 0.632** | | 0.512* | | -0.436* | | | | |  | | | 0.861** | | 0.875**(0.281) | | | -0.920** | |  | | -0.831** | |  | | 0.413(0.423) | | 0.526(0.537) |
|  | 186tm | 21 | | 0.545* | | 0.523* | | -0.540* | | | | |  | | | 0.752** | | 0.850**(0.184) | | | -0.930** | |  | | -0.755** | |  | | 0.433(0.444) | | 0.549(0.561) |
